# The amyloid PET status in Primary Progressive Aphasia

**DOI:** 10.1002/alz.088485

**Published:** 2025-01-09

**Authors:** Soichiro Shimizu, Ryo Yamamoto, Shoya Inagawa, Naoto Takenoshita

**Affiliations:** ^1^ Tokyo Medical University, Tokyo Japan

## Abstract

**Background:**

Primary Progressive Aphasia (PPA) is a clinical and symptomatic concept due to neurodegenerative diseases which language symptom was dominant from early clinical stage. PPA is categorized into 3 subtypes such as Progressive non‐fluent aphasia (PNFA), Semantic dementia (SD), and logopenic progressive aphasia (LPA). In this study, we compared the differences of PPA subtypes depend on amyloid PET results.

**Method:**

We recruited PPA patients who underwent standard aphasia test (SLTA) as Japanese aphasia test, cerebrospinal fluid AD biomarker, and amyloid PET. We divided into Amyloid PET positive (A(+) PPA) and negative (A(‐) PPA) group. then we compared the characterization of SLTA results and CSF biomarker between two groups.

**Result:**

A total of 15 patients were included. There were 7 patients in A (+) PPA groups (2 males and 5 females, age: 65.3±8.3 years), and 8 patients in A (‐) PPA group (6 males and 2 females, age: 66.6±11.2 years). Regarding cerebrospinal fluid AD biomarkers, a significant decrease in Aβ42/40 (p < 0.01) and significant elevation in P‐tau (p<0.01) was observed in the A (+) PPA group compared to the A (‐) PPA group. In SLTA, the A (+) PPA had LPA: 57% and transcortical sensory aphasia: 29%. on the other hand, A (‐) PPA group had PNFA: 38% and SD: 50%.

**Conclusion:**

This study showed there is the possibility of SLTA may be useful to predict AD pathology in patients with PPA.

